# miR-199a-3p displays tumor suppressor functions in papillary thyroid carcinoma

**DOI:** 10.18632/oncotarget.1830

**Published:** 2014-03-16

**Authors:** Emanuela Minna, Paola Romeo, Loris De Cecco, Matteo Dugo, Giuliana Cassinelli, Silvana Pilotti, Debora Degl'Innocenti, Cinzia Lanzi, Patrizia Casalini, Marco A. Pierotti, Angela Greco, Maria Grazia Borrello

**Affiliations:** ^1^ Molecular Mechanisms Unit, Department of Experimental Oncology and Molecular Medicine, Fondazione IRCCS Istituto Nazionale dei Tumori, Milan, Italy; ^2^ Functional Genomics Core Facility, Fondazione IRCCS Istituto Nazionale dei Tumori, Milan, Italy; ^3^ Molecular Pharmacology Unit, Department of Experimental Oncology and Molecular Medicine, Fondazione IRCCS Istituto Nazionale dei Tumori, Milan, Italy; ^4^ Department of Pathology, Fondazione IRCCS Istituto Nazionale dei Tumori, Milan, Italy; ^5^ Molecular Targeting Unit, Department of Experimental Oncology and Molecular Medicine, Fondazione IRCCS Istituto Nazionale dei Tumori, Milan, Italy; ^6^ Scientific Directorate, Fondazione IRCCS Istituto Nazionale dei Tumori, Milan, Italy

**Keywords:** miR-199a-3p, Papillary thyroid carcinoma, microRNA, RET/PTC, MET, oncogene

## Abstract

Thyroid cancer incidence is rapidly increasing. Papillary Thyroid Carcinoma (PTC), the most frequent hystotype, usually displays good prognosis, but no effective therapeutic options are available for the fraction of progressive PTC patients. *BRAF* and *RET/PTC* are the most frequent driving genetic lesions identified in PTC. We developed two complementary *in vitro* models based on *RET/PTC1* oncogene, starting from the hypothesis that miRNAs modulated by a driving PTC-oncogene are likely to have a role in thyroid neoplastic processes. Through this strategy, we identified a panel of deregulated miRNAs. Among these we focused on miR-199a-3p and showed its under-expression in PTC specimens and cell lines. We demonstrated that miR-199a-3p restoration in PTC cells reduces MET and mTOR protein levels, impairs migration and proliferation and, more interesting, induces lethality through an unusual form of cell death similar to methuosis, caused by macropinocytosis dysregulation. Silencing MET or mTOR, both involved in survival pathways, does not recapitulate miR-199a-3p-induced cell lethality, thus suggesting that the cooperative regulation of multiple gene targets is necessary. Integrated analysis of miR-199a-3p targets unveils interesting networks including HGF and macropinocytosis pathways. Overall our results indicate miR-199a-3p as a tumor suppressor miRNA in PTC.

## INTRODUCTION

Thyroid cancer (TC) is the most common endocrine malignancy, with a continuously increasing incidence over the past few decades [1-3]. TC, derived from thyroid follicular cells, includes papillary thyroid carcinoma (PTC), follicular thyroid carcinoma (FTC), poorly differentiated (PDTC) and undifferentiated anaplastic (ATC) carcinoma [4]. PTC, the most prevalent histotype, is well-differentiated and usually associated with a good prognosis and therapeutic response; nevertheless, about 10% of patients present with recurrences and distant metastases. The prognosis for these patients is very poor, as they have no therapeutic options [5;6].

MicroRNAs (miRNAs), endogenous RNA molecules of 19-25 nt, are post-transcriptional regulators of gene expression acting via translational blockade or transcript degradation [7]. MiRNAs involvement in human cancer is well established: they represent a new level of gene regulation and a potential tool as diagnostic, prognostic and therapy response biomarkers [8]. The main miRNAs alteration in cancer cells is abnormal expression. Accordingly, recent reports defined miRNA profiles that could discriminate between PTC and normal thyroid tissue, suggesting their value as diagnostic markers [9-13]

However, while the aberrant expression of specific miRNAs in PTC is established (as for miR-221/222 and miR-146), the involvement of other miRNAs is still unclear [14]. Even though interesting functional studies have addressed the role of specific miRNAs in thyroid carcinogenesis, the mechanisms leading to miRNAs deregulation in neoplastic thyroid cells and the consequences of their deregulation are far from being fully elucidated [15-18].

In PTC, alternative genetic lesions, including *RET* or *TRK* rearrangements and *BRAF* or *RAS* mutations [19], have been identified as driving oncogenes in approximately 70% of cases. By exploiting these oncogenes, it is possible to generate reliable *in vitro* models of PTC: through this approach we previously identified a set of genes, induced by *RET/PTC1* in thyrocytes, whose expression has been validated in PTC specimens [20].

In the present work, we have used the same cell model to establish miRNA expression profiles regulated by *RET/PTC1*. In addition, we developed a second model, based on TPC1 cells treated with the tyrosin kinase (TK) inhibitor RPI-1 able to counteract the activation of the endogenous *RET/PTC1* oncogene [21]. Through these cellular models, we identified genes and miRNAs concordantly regulated by the *RET/PTC1* oncogene. These latter include miRNAs already known as differentially expressed in PTC clinical samples as well as additional miRNAs, comprising the miR-199 family. MiR-199a is a phylogenetically conserved miRNA whose precursors miR-199a-1 and miR-199a-2 map in human genome to different loci, respectively on chromosome 19 and on chromosome 1 (Supplementary Fig. S1A). From both hairpin precursors, two mature sequences are produced: miR-199a-5p and miR-199a-3p. MiR-199a-2 is also reported as a member of miR-199a-2/214 cluster [22].

In this work, we have demonstrated that miR-199a-3p is under-expressed in human PTC specimens and in PTC-derived cell lines, and displays tumor suppressor functions in papillary thyroid carcinoma. MiR-199a-3p is able to reduce MET and mTOR protein levels, MET-dependent migration, invasion and proliferation. Most interestingly, miR-199a-3p induces lethality in PTC cells through a non-apoptotic form of cell death, similar to methuosis, recently described as caused by macropinocytosis excess [23].

## RESULTS

### *In vitro* modeling of papillary thyroid carcinoma: *RET/PTC1* oncogene-dependent miRNA and coding gene expression profiles

**Figure 1:**
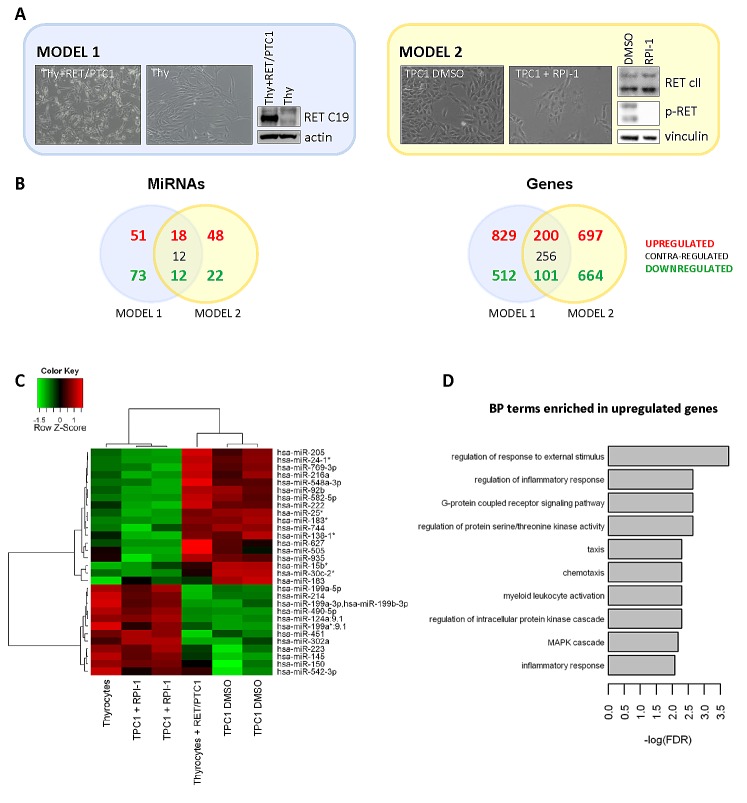
Micro-RNA and gene expression profiles of *in vitro* PTC models based on *RET/PTC1* oncogene (A) *In vitro* cell models used to identify RET/PTC1-regulated miRNAs and genes in thyroid cells. Model 1: *RET/PTC1*-infected human thyrocytes vs parental thyrocytes; Model 2: TPC1 cells DMSO-treated vs TPC1 cells treated with the RET inhibitor RPI-1. Representative phase-contrast micrographs of thyroid cells from the two models (magnification 100x) and the corresponding expression of RET/PTC1 oncoproteins: model 1 exogenous RET/PTC1-isoform 9 (analysed with RET C19 primary antibody) and model 2 endogenous RET/PTC1-isoforms 9 and 51 (analysed with RET common II primary antibody) with their Tyr-phosphorylation are shown. For biochemical analysis cell extracts were analyzed with the indicated antisera using actin or vinculin as protein loading control. (B) Venn diagrams showing miRNAs (absolute fold change > 2.5 for model 1; absolute fold change > 2.5 and FDR < 0.1 for model 2) and genes (absolute fold change > 1.5 and FDR < 0.005) commonly differentially expressed between the two cell models. (C) Heat Map representing color-coded expression levels of the 30 miRNAs commonly differentially expressed and concordantly regulated between the two models. For the model 1 biological replicates couldn't be generated. Thyrocytes expressing *RET/PTC1* oncogene were compared to parental thyrocytes performing a fold-change analysis filtering out miRNAs with an expression value < 8 in order to reduce the risk of false positive hits. For the model 2 biological triplicates were generated by independent treatments and RNA extractions. Two samples (one treated and one control) were excluded due to low quality profiles. Signal intensities averaged between biological replicates for DMSO treated cells were normalized to the average signal of RPI-1 -treated cells. (D) Barplot showing significant Gene Ontology terms of the Biological Process domain significantly over-represented (FDR < 0.01) in the list of commonly upregulated genes between the two models.

To generate *in vitro* models of papillary thyroid carcinoma (PTC), two cell systems were set up: primary human thyrocytes exogenously expressing the *RET/PTC1* oncogene vs parental thyrocytes (model 1) [20], and TPC1 cells (PTC-derived cell line harbouring endogenous *RET/PTC1*) solvent-treated vs TPC1 cells treated with the TK inhibitor RPI-1 (model 2) [21]. The cell morphology and the corresponding expression of RET/PTC1 oncoproteins in the two models are shown (Figure 1A).

Microarray miRNA and mRNA expression profiles obtained from both models (details in Materials and Methods) were compared: we identified a total of 30 miRNAs and 301 coding genes concordantly regulated accordingly with the presence of an active RET/PTC1 oncoprotein (Venn diagrams, Figure 1B). Overlapping miRNAs (Heatmap, Figure 1C) interestingly include: miR-222, whose over-expression is considered a hallmark of thyroid malignancy; miR-205, sporadically reported as over-expressed in thyroid carcinomas with respect to non-neoplastic thyroid [24]; miR-451, under-expressed in PTC [15;25;26] and other miRNAs poorly or not investigated in papillary thyroid carcinoma.

Among these, the miR-199 family, including mature miR-199a-5p, miR-199a-3p as well as miR-214, that clusters with the precursor mir-199a-2 (Supplementary Fig.S1A), stands out as significantly down-regulated by *RET/PTC1*. Interestingly, miR-199a-3p down-regulation is confirmed by two different probes (hsa-miR-199a-3p/b-3p and hsa-miR-199a*:9.1) present on the utilized Illumina miRNA platform (Supplementary Fig. S1B).

The analysis of mRNAs commonly and concordantly regulated in the two cell models (Figure 1D) highlights the up-regulation of genes involved in intracellular signalling, inflammation and chemotaxis many of which reported over-expressed in PTC with respect to non-neoplastic thyroid (e.g. *CXCL1, CXCL2, IL8, PLAU, PLAUR, MET, EREG* [20;27-30]).

**Figure 2:**
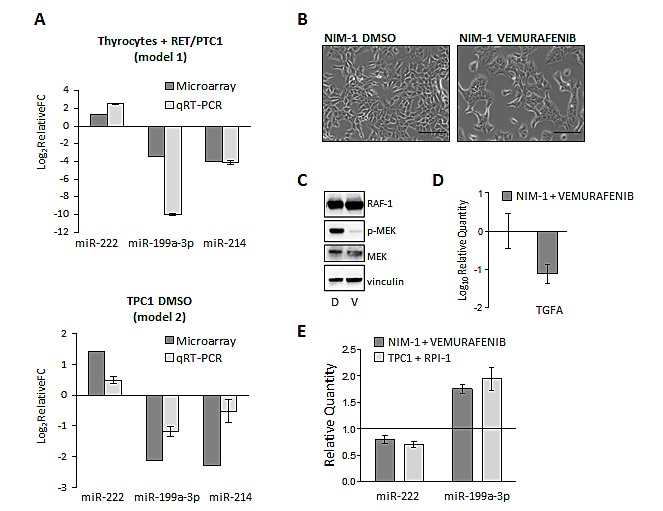
MiR-199a-3p is modulated by *RET/PTC1* and *BRAF-V600E* oncogenes (A) qRT-PCR validation of model 1 and model 2 microarray data for selected miRNAs. The expression levels of miR-222, miR-199a-3p and miR-214 were measured by qRT–PCR in cells from the two models. MiRNA levels, both for microarray and qRT-PCR data, were expressed as relative Fold Change (FC) of Thyrocytes expressing *RET/PTC1* vs parental thyrocytes (model 1) and of TPC1 DMSO vs TPC1 RPI (model 2). (B) Effect of BRAF-V600E inhibition on the expression levels of selected miRNAs. Representative phase-contrast micrographs of NIM-1 cells exposed to vehicle DMSO or vemurafenib for 20h are shown (scale bar 100 μm). (C) Cell extracts from NIM-1 treated with DMSO (D) or vemurafenib (V) were analyzed with the indicated antisera. Vinculin is shown as protein loading control. (D) The expression level of TGFA was evaluated by qRT-PCR in NIM-1 cells treated with DMSO or vemurafenib. Data are normalized to the value of NIM-1 DMSO. (E) MiR-199a-3p and miR-222 expression levels were evaluated by qRT-PCR in NIM-1 and in TPC1 cells treated with vemurafenib or RPI-1 and DMSO. Data are shown relative to the miRNA levels of the corresponding DMSO treated control cells, represented as solid line at the value 1. All qRT-PCR results were presented as mean ± SEM from triplicate assays and normalized to let-7a used as endogenous control

Microarray expression data of three selected miRNAs among those commonly deregulated in the two cell models has been validated by qRT-PCR (Figure 2A). We confirmed that the expression of *RET/PTC1* (Thyrocytes+*RET/PTC1*, upper panel) or the presence of the active RET/PTC1 oncoprotein (TPC1 DMSO treated, lower panel) causes miR-222 up-regulation and miR-199a-3p and miR-214 down-regulation.

Furthermore, to investigate if selected miRNAs might be regulated also by *BRAF-*V600E, the most frequently identified oncogene in PTCs [19], we treated the PTC-derived NIM-1 cells, harboring *BRAF-*V600E mutation, with the BRAF inhibitor vemurafenib (Figure 2B) and analyzed the expression levels of miR-222 and miR-199a-3p by qRT-PCR

Firstly, we verified that in NIM-1 cells vemurafenib treatment almost abolishes MEK phosphorylation downstream to BRAF (Figure 2C) and reduces cell proliferation (not shown), as expected [31]. In addition, as control of vemurafenib effect, the down-regulation of *TGFA* gene is shown (Figure 2D), in agreement with our previous observations that *BRAF*-V600E-positive PTCs display elevated TGFA levels and that in NIM-1 cells TGFA expression is dependent on RAF/MEK/ERK pathway [27]. Then, we found that in NIM-1 cells the vemurafenib treatment is able to decrease miR-222 and increase miR-199a-3p expression, as observed for the RPI-1 treatment in TPC1 cells (Figure 2E). Thus, miR-199a-3p might be regulated also by the *BRAF*-V600E oncogene.

Moreover, accordingly with previous reports that indicate *MET* as a target gene of miR-199a-3p in different cell types [32;33], our genes and miRNAs microarray data indicated an opposite expression pattern for MET and miR-199a-3p in both the above described RET/PTC1 models (data not shown). Whereas MET is reported over-expressed in about 70% of PTC surgical specimens [28], miR-199a-3p has not been commonly identified as a PTC-associated miRNA and no functional studies in PTC-derived cells have been performed. Thus, we focused on miR-199a-3p and on its possible role in thyroid carcinogenesis.

### Under-expression of miR-199a-3p in papillary thyroid carcinomas

**Figure 3:**
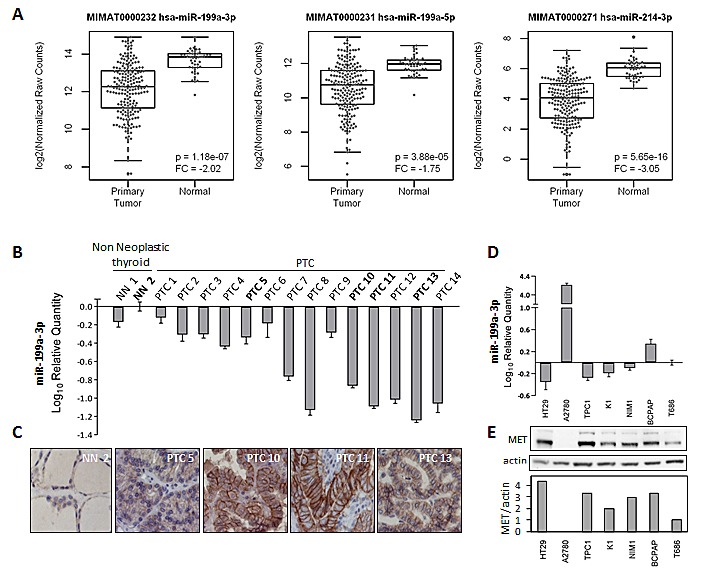
MiR-199a-3p and MET expression levels in PTC tissue samples and in PTC derived cell lines (A) Boxplot showing the expression levels of hsa-miR-199a-3p, hsa-miR-199a-5p and hsa-miR-214-3p (previous ID hsa-miR-214, see Supplementary Figure.1) in papillary thyroid carcinoma (n=203) and in normal (n=44) samples. miRNA-Seq and clinical data for papillary thyroid carcinoma samples were downloaded from The Cancer Genome Atlas (TCGA) data portal. Samples with an undefined histological type were excluded. Expression values, calculated as reads per million miRNA mapped were log2 transformed and p-values were calculated by binomial negative distribution test. FC: Fold Change of papillary thyroid carcinomas vs normal samples (B) qRT-PCR analysis of miR-199a-3p expression levels in PTC surgical samples. (C) FFPE sections derived from selected PTC samples were analyzed for MET protein expression by IHC. One representative non-neoplastic thyroid and four PTC cases are shown (magnification 100x). (D) qRT-PCR analysis of miR-199a-3p expression levels in PTC cell lines and in the normal thyroid cells T686. HT-29 and A2780 cell lines were used as miR199a-3p low and high expression control respectively. Data are normalized on the T686 value, considered as baseline. (E) Western blot analysis of MET protein in the same cells panel and the corresponding densitometric quantification of MET normalized to the level of loading control protein (actin). All qRT-PCR data were presented as mean ± SEM from triplicate assays and normalized to U6 small nucleolar RNA used as endogenous control.

The expression of miR-199a-2/214 cluster members was investigated in PTC surgical specimens. Firstly, the public dataset “The Cancer Genome Atlas” (TCGA) was interrogated for the expression of the precursors and the matures miR-199a-3p, miR-199a-5p and miR-214 in thyroid samples, including 203 PTCs and 44 normal thyroids. TCGA data indicate that, although heterogeneous, both precursors (data not shown) and matures miR-199a-3p, miR-199a-5p and miR-214 are significantly under-expressed in PTCs compared with normal thyroids (Figure 3A).

To further corroborate miR-199a-3p result, a case list of 14 PTC specimens (clinical-pathological features available in Supplementary Table 1) collected in our Institute, was assessed for miR-199a-3p expression by qRT-PCR, and a subset of cases (matched FFPE) was analyzed for MET expression by immunohistochemistry (IHC). In our PTC case list miR-199a-3p was under-expressed compared with non-neoplastic thyroids (Figure 3B), confirming TCGA data, and, as expected, MET expression was medium/high in PTCs and very low/absent in non-neoplastic thyroids (Figure 3C).

Subsequently, the expression of miR-199a-3p was analyzed by qRT-PCR in the PTC-derived cell lines TPC1 (harboring the *RET/PTC1* oncogene and already exploited for model 2), K1, NIM-1, and BCPAP (all harboring the *BRAF*-V600E oncogene), as well as in T686 cells (non neoplastic human thyrocytes), in HT-29 and A2780 cell lines (included respectively as miR-199a-3p low and high expression control [32]). All the analyzed thyroid-derived cells, both tumoral and non neoplastic cells, express low miR-199a-3p levels, similar to the HT-29 low expression control. In addition, among the PTC-derived cell lines, all but one (BCPAP cells) show under-expression of miR-199a-3p compared with the non neoplastic control T686 (Figure 3D).

Immunoblot analysis of the same cell panel confirmed MET protein over-expression in all PTC cell lines, suggesting a possible inverse relationship miRNA/target gene (Figure 3E).

As BCPAP cell line may be representative of the small fraction of PTCs expressing miR-199a-3p levels similar to or higher than the non-neoplastic thyroids, as shown in the TCGA data, only TPC1, NIM-1 and K1 cells, representative of the majority of PTC surgical specimens, were selected for the next functional experiments.

### MiR-199a-3p impairs proliferation and migration in PTC-derived cell lines

To unveil the biological effects of miR-199a-3p in thyroid carcinoma cells, the PTC-derived cell lines TPC1, NIM-1 and K1, all under-expressing miR-199a-3p (Figure 3D), were transfected with a miR-199a-3p synthetic miRNA mimic (hereafter indicated as miR-199a-3p). In TPC1 cells we verified by qRT-PCR that following transfection miR-199a-3p is exogenously expressed, results stable from 24 to at least 72 hours post-transfection and is able to reduce MET protein maximally at 72 hours after transfection (Supplementary Fig.S2). The Western blot analysis of MET protein level at 72 hours has been exploited as “read-out” of miR-199a-3p exogenous expression and function in the all hereafter reported transfection experiments.

**Figure 4:**
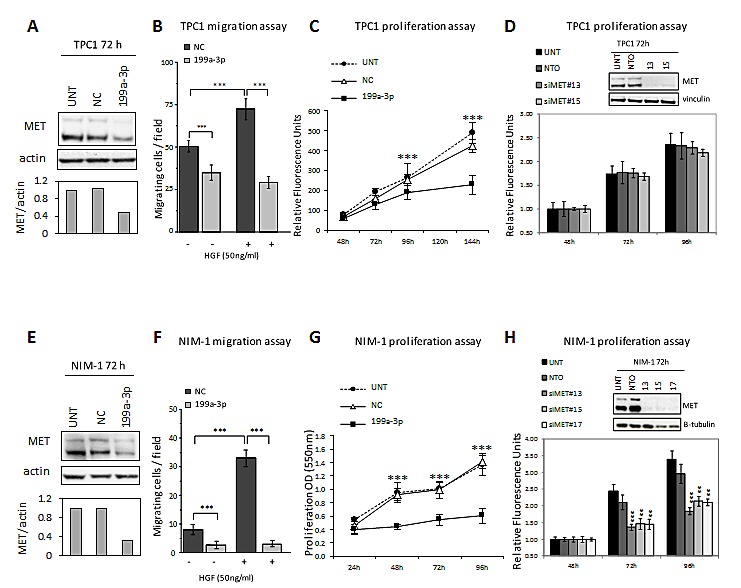
Functional effects of miR-199-a-3p restoration and MET silencing in TPC1 and NIM-1 cells (A, E) Both cell lines were either left untransfected (UNT) or transfected with miR-199a-3p (199a-3p) or Negative-Control (NC). 72h post-transfection total cell lysates were analysed by immunoblotting for MET expression using actin as loading control. MET protein levels were quantified by densitometric analysis and represented relative to the value of the untreated control cells. (B, F) Migration capability was evaluated 48h post-transfection in Transwell chambers in absence or presence of HGF. Assays were performed in triplicate, cells were counted in adjacent fields (n=10); data are reported as average cell number per field ± SD. (C, G) Proliferation assays were performed as described in Materials and Methods. In NIM-1 cells transfected with miR-199a-3p or Negative-Control cell growth was evaluated by sulforhodamine B (SRB) colorimetric assay at the indicated times. (D, H) For MET silencing experiments both cell lines were left untransfected (UNT) or transfected with MET-specific siRNAs (siMET) or Non-targeting control (NTO). MET knockdown was confirmed by Western blot 72h post-transfection, using β-tubulin or vinculin as loading control. Statistical significance was determined via Student's t-test; ** P value<0.001 *** P value<0.0001.

Functional analyses show that miR-199a-3p restoration in TPC1 and NIM-1 cells, confirmed by MET protein reduction (Figure 4A and E), significantly impairs the migration ability either in absence or presence of the MET ligand HGF (Figure 4B and F) and proliferation in both cell lines (Figure 4C and G). Moreover, miR-199a-3p significantly decreases the NIM-1 cells invasiveness in Matrigel layer (Supplementary Fig.S4A).

To define the possible role of MET in the phenotypes induced by miR-199a-3p we used a selective gene silencing approach. As shown, MET specific siRNAs almost abolish MET expression in both TPC1 and NIM-1 cell lines (Figure 4D and H) and significantly reduce the proliferation of NIM-1, but not of TPC1 cells. We further demonstrated that MET silencing reduces migration in TPC1 cell line (data not shown).

### MiR-199a-3p induces non-apoptotic cell death in K1 cells

**Figure 5:**
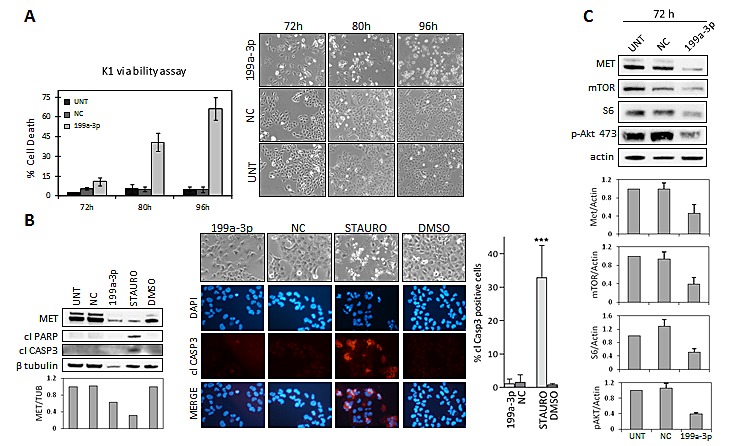
miR-199-a-3p-induced cell death in K1 cell line (A) Viability based on PI staining was assessed in K1 cells untransfected (UNT) or transfected with miR-199a-3p (199a-3p) or Negative-Control (NC) at the indicated times. Data are reported as mean ± SD of triplicate counts per condition for at least three independent experiments. Right panel: representative images of K1 cells at the same time points (LEICA inverted microscope, magnification 100x) (B) K1 cells transfected as in (A) collected 80h post-transfection and K1 cells exposed for 24 hours to vehicle or to staurosporine for apoptosis induction were analysed by immunoblotting with the indicated antisera (left panel). Caspase activation was also assessed in the same cells by immunofluorescence with anti-cleaved caspase-3 specific antibody. IF images acquired with a Nikon Eclipse TE2000-S microscope (magnification 200x); above representative images at the moment of collection (LEICA inverted microscope, magnification 100x). The corresponding percentage of cleaved-caspase 3 positive cells (right panel) was calculated per field relative to the total cells number, determined by DAPI staining. Results represent mean ± SD of triplicate counts of at least 5 fields randomly selected per condition. ***P < 0.0001 statistical significance determined via Student's t-test. (C) Immunoblot analysis of K1 total cell lysates extracted 72h post-transfection. Protein levels were quantified and normalized to the level of loading control protein (actin). Data, presented relative to the value of K1 untreated control cells, are shown as mean expression value ± SD for 3 independent experiments.

Analogous experiments were performed in K1 cells, carrying *BRAF*-V600E and *PIK3CA*-E542K mutations, and also in this cell line miR-199a-3p effective over-expression following transfection was confirmed by MET protein levels reduction (Figure 5C).

K1 cells transfected with miR-199a-3p exhibited different morphology compared with the control cells, displaying high cytoplasmic vacuolization followed by cell rounding, progressive detachment from the plate and cells disruption, as suggested by the presence of floating debris. Viability assay confirmed that, starting from 72 hours up to 96 hours, miR-199a-3p-transfected K1 cells massively died (Figure 5A). To determine the nature of the observed cell death we first investigated apoptosis markers. K1 cells transfected with miR-199a-3p do not exhibit the typical features of apoptosis such as nuclear hypercondensation/fragmentation, apoptotic bodies, cleaved Caspase3 or cleaved PARP, that are instead evident in K1 cells treated with the apoptosis inducer staurosporine (Figure 5B).

Thus to further characterize the effects of miR-199a-3p in K1 cells Western blot analyses were performed. As shown in Figure 5C, miR-199a-3p restoration reduces mTOR, an experimentally validated target of miR-199a-3p in liver cell context [33], S6, a downstream element in mTOR pathway, and phospho-AKT protein levels, suggesting that miR-199a-3p regulates AKT/mTOR pathway.

**Figure 6:**
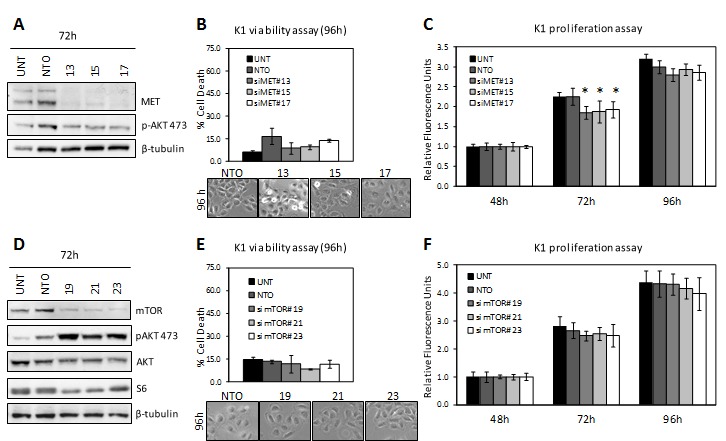
Knockdown of MET and mTOR in K1 cells (A, D) K1 cells were left untransfected (UNT) or transfected with specific siRNAs (siMET or si mTOR) or Non-targeting control (NTO). Knockdown was confirmed by Western blot 72h post-transfection, following the membranes were probed with the indicated antisera; β-Tubulin as loading control. (B, E) Viability, assessed as described in Figure 5A, was evaluated at 96h post-transfection. Below are displayed representative images of K1 transfected cells at the same time point (magnification 100x). (C, F) Proliferation analysis was performed as described in Materials and Methods. Statistical significance was determined via Student's t-test; *P value<0.05

Thus, to assess a possible role of MET or mTOR, both involved in cell survival and negatively regulated by miR-199a-3p, in K1 lethality we silenced these genes (Supplementary Fig.S3A-B). As shown in Figure 6, although the respective siRNAs knockdown the expression of MET or mTOR more efficiently than miR-199a-3p (Figure 6A and D), neither cause K1 cell death (Figure 6B and E). Only MET silencing, decreasing AKT phosphorylation, impairs K1 cell proliferation (Figure 6C), as observed in NIM-1 cells (Figure 4B and Supplementary Fig.S3B). At variance, mTOR knockdown, triggering a feedback loop, induces AKT over-activation and does not affect proliferation (Figure 6F). Moreover, S6 protein decrease observed in miR-199a-3p-transfected K1 cells was confirmed in mTOR-silenced K1 cells, suggesting that S6 down-regulation is more probably due to feedback regulation than to the direct action of miR-199a-3p.

MiR-199a-3p-induced lethality observed in K1 cells was further confirmed in NIM-1 cells. Indeed, by using a more efficient transfection method we found that massive cell death occurs in K1 cells within 48 hours and in NIM-1 cells within 96 hours post transfection (Supplementary Fig.S4B).

### MiR-199a-3p induces macropinocytosis in K1 cells

**Fig.7:**
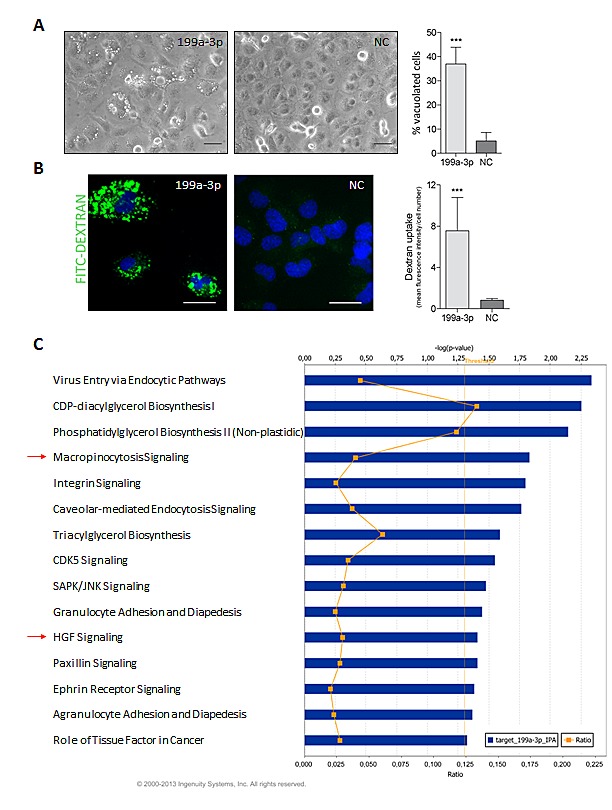
miR-199-a-3p induces macropinocytosis in K1 cell line (A) Representative images of K1 cells transfected with miR-199a-3p (199a-3p) or Negative-Control (NC) 72h post-transfection. (LEICA inverted microscope, scale bar 50 μm). The percentage of vacuolated cells was determined at 72h post-transfection by counting at least 150 cells per condition using a threshold of two or more vacuoles per cell for a positive score. Data are presented as mean ± SD for 3 independent experiments. (B) Representative images of K1 transfected cells labeled with FITC-Dextran. Images were captured with identical exposure settings with a Nikon Eclipse E1000 microscope (scale bar 100 μm). Quantification of Dextran uptake was calculated by Image-Pro Plus 7.0.1 software by measuring the green fluorescence mean intensity normalized to the number of cells, determined by DAPI staining, per each field. At least 5 fields were randomly selected and scored per condition. Barplot displays mean ± SD for 2 independent experiments. ***P < 0.0001 statistical significance determined via Student's t-test. (C) miR-199a-3p targets Top Canonical Pathway. The gene list of inversely correlated targets of miR-199a-3p (Supplementary Figure S6) were analysed by IPA using a core analysis. In the bar chart are displayed significant Canonical Pathways (Threshold p-value <0.05) and the corresponding value of ratio, calculated by dividing the number of analyzed genes found to map in a specific pathway by the total number of genes that constitute that pathway.

Our initial observations revealed the presence of numerous phase-lucent cytoplasmic vacuoles in miR-199a-3p-transfected K1 cells (Figure 7A). Maltese group described an uncommon mechanism of non-apoptotic cell death in which vacuoles accumulate in cell cytoplasm [23]. The authors demonstrated that these vacuoles are enlarged macropinosomes and that this form of cell death, named methousis, is characterized by macropinocytosis hyperstimulation.

Thus, to further explore the type of cell death induced by miR-199a-3p, we investigated the possible involvement of macropinocytosis. By testing the ability of cells to internalize the extracellular fluid-phase tracer FITC-dextran, an established marker of macropinocytosis, we found that K1 cells expressing miR-199a-3p rapidly incorporate dextran, suggesting that miR-199a-3p hyper-stimulates macropinocytosis in these cells (Figure 7B). Moreover, by a dual-marker assay, we confirmed, as previously described in RAS-activated glioblastoma cells [23], that FITC-dextran and Lysotracker do not co-localize, suggesting that also in thyroid cell context macropinocytotic vacuoles are distinct from lysosomes (Supplementary Fig.S5A). A further evidence that the vacuoles are macropinosomes, was obtained by EIPA treatment, known to inhibit macropinocytosis [34;35]: EIPA significantly reduced the percentage of vacuolated cells in miR-199a-3p expressing K1 cells (Supplementary Fig.S5B).

To unveil genes regulated by miR-199a-3p in PTC, we integrated model-1 gene expression data with the i*n silico* predicted miR-199a-3p gene targets (details in Supplementary material and methods). Interestingly, Ingenuity Pathway Core Analysis (IPA) of the obtained list (Supplementary Fig.S6) revealed, among significant Canonical Pathways, HGF and macropinocytosis pathways, both experimentally demonstrated as regulated by miR-199a-3p in PTC cell lines (Figure 7C).

## DISCUSSION

In this study we exploited *in vitro* models of PTC to unveil a novel tumor suppressor role of miR-199a-3p in this neoplasia.

Aberrant deregulation of microRNA expression has been well documented in numerous human tumors and many efforts have been undertaken to understand the regulatory mechanisms that govern and in turn are controlled by these small molecules. MiRNAs act as oncogenes or tumor suppressor when they positively or negatively regulate key genes involved in cellular processes associated to tumor genesis or progression [36]. At the same time it has been reported that several known oncogenic pathways promote carcinogenesis by a fine tuning of miRNAs expression, obtained through different mechanisms as regulation of transcription factors [37-39].

In the last decades several studies have compared global miRNA expression in PTCs versus non neoplastic thyroid tissues exploiting high throughput analyses, nevertheless due to the great variability among the different experimental sets and protocols, there is agreement only about few miRNAs significantly and reproducibly deregulated in PTCs such as miR-146, miR-181b, miR-221 and miR-222 [14]. Indeed, individual genetic variability as well as the heterogeneity of the tumors may hamper the identification of functionally relevant deregulated miRNAs. The generation of appropriate cell model systems might help to “read” expression profile data [40-42].

PTC is unique among epithelial tumors, as apparently develops in one step without a benign pre-invasive counterpart, and the generation of the *RET/PTC* oncogene is a recognized early and driving genetic event in the pathogenesis of a fraction of PTCs [43-46].

Thus, we hypothesized that miRNAs regulated by oncogenes causally related to PTC development, such as *RET/PTC1*, are likely to have a role in neoplastic transformation of thyroid cells, and that oncogene-independent pathways may drive convergent miRNAs de-regulation. Following this strategy, we developed two complementary cell models based on *RET/PTC1* oncogene. The first model, human thyrocytes exogenously expressing the *RET/PTC1*, has been already useful in the identification of a gene set involved in inflammation/invasion validated in surgical specimens [20]. The second model consisted of TPC1 cells (harbouring endogenous *RET/PTC1*) treated with the TK inhibitor RPI-1 [21]. We hypothesized that miRNAs deregulated in both models are likely to have a role in PTC cancerogenesis.

Here we identified a list of genes and miRNAs commonly and concordantly regulated by *RET/PTC1* in the two cell models. These latter include the upregulated miR-222, reported among miRNAs reproducibly over-expressed in PTC surgical samples and involved in the cell cycle regulation in PTC cells [9;14;15], as well as miRNAs whose role in PTC is not known. Among these, miR199a-2/214 cluster, comprising miR-199a-3p, miR199a-5p and miR-214, emerged as down-regulated by the RET/PTC1 oncogene. Moreover, we showed that also *BRAF*-V600E oncogene likely regulates miR-199a-3p.

MiR-199a-3p has been shown to have different expression patterns and to play opposite roles in different human tumors: it promotes proliferation and metastasis in gastric cancer and it is related to poor prognosis and tumor progression in ovary cancer, but it displays anti-proliferative/tumor suppressor functions in human hepatocellular carcinoma, osteosarcoma, testicular germ cell tumors, and bladder cancer (rev in [22]).

In thyroid carcinomas few studies described miR-199a-3p under-expression in tumor histotypes different than PTC [25;47]. MiR-199a-3p has not been previously recognized among the relevant miRNAs signature associated to PTC. To date, only one paper reported in PTC a statistically significant down-regulation of the precursor miR-199a-1, based on miRNA expression profile [48]. Nevertheless, neither details about the corresponding mature miRNA levels nor qRT-PCR validation nor functional studies are reported in the above-mentioned paper. Here we showed that the mature miR-199a-3p is under-expressed in PTC surgical samples compared to non-neoplastic thyroid, assessed by meta-analysis in a large cohort and by qRT-PCR in our small case list, and in the majority of the analyzed PTC-derived cell lines relative to immortalized thyrocytes.

To our knowledge no functional studies for this miRNA are reported in PTC. The only work that explores by functional experiments the role of one miR-199a-2/214 cluster member in thyroid context, analysed the miR-199b-5p in follicular thyroid cancer (FTC) [17]. Thus we focused on miR-199a-3p and to investigate its functional role we restored miR-199a-3p expression in three PTC-derived cell lines and analysed several phenotypes, including proliferation, invasion and cell death.

In a different cellular context miR-199a-3p was reported to target MET [32;33;49], a gene over-expressed in the majority of PTC [28]. We have previously demonstrated that RET/PTC1-driven neoplastic transformation and pro-invasive phenotype of human thyrocytes involve MET induction [30]. Here we demonstrated that miR-199a-3p down-regulates MET protein also in PTC-derived cell lines. Remarkably, miR-199a-3p forced expression in these cells induced strong biological effects including proliferation, migration and invasion inhibition, and even cell death. The strongest effect was achieved in K1 cells (harbouring *BRAF*-V600 and PIK3CA-E542K activating mutations), which die massively 80-96 hours after miR-199a-3p restoration. Moreover, a consistent fraction of NIM-1 cells die after the transfection of miR-199a-3p by using a more efficient transfection system. Interestingly, miR-199a-3p does not induce cell lethality when exogenously expressed in T686 non neoplastic thyroid cell line (our data not shown). Of note, the transfection of miR-199a-3p was reported to induce caspase-independent cell death in several cancer cell lines from lung, breast, and prostate carcinomas [50]. As we have shown that miR-199a-3p down-regulates MET and mTOR proteins in PTC cells, as previously demonstrated in other cell context [32;33], we then silenced MET or mTOR to assess their possible role in the miR-199a-3p induced lethality.

We demonstrated that MET is partially responsible for miR-199a-3p-triggered phenotypes as proliferation and migration, whereas mTOR silencing is less effective, probably due to the complex network of feedbacks regulating this pathway. However, neither MET nor mTOR silencing caused cell lethality. Thus, restoring miR-199a-3p in PTC cells seems to have stronger anti-neoplastic effects than each of its identified targets.

The cell death induced by miR-199a-3p is unusual and interesting, although the exact mechanism remains to be fully characterized. It does not present the typical features of apoptosis such as apoptotic nuclei, PARP cleavage or caspases activation. At variance, miR-199a-3p-transfected cells seem to behave like glioblastoma cells, in which the expression of activated RAS induces an unusual form of cell death, named methuosis that is caused by macropinocytosis dysregulation [23]. Macropinocytosis is a physiologic process by which cells internalize extracellular fluid into vesicles defined macropinosomes. However, as described by Maltese group, hyperstimulation of macropinocytosis together with defects in the endocytic vesicle trafficking and recycling, could results in accumulation of progressively large vacuoles, leading to disruption of cellular membrane integrity and cells death [51].

To our knowledge, this death type, associated with the RAS-RAC pathway deregulation, has not been previously causally related to a miRNA. As cancer cells are often resistant to apoptosis, identifying genetic elements driving non-apoptotic cell death is a potentially interesting therapeutic tool. It is conceivable that the observed lethal phenotype is due to miR-199a-3p action on multiple targets and is mediated by networks including MET, mTOR and molecules involved in macropinocytosis hyperstimulation, as suggested by functional analyses and IPA tool. Among miR-199a-3p targets predicted by integrated analysis, there are indeed genes involved in macropinocytosis, such as ABI1 [52], and other genes worthy of further investigation due to their oncogenic relevance in PTC (listed in supplementary informations). These include C8orf4, also known as thyroid cancer 1 (TC-1) [53], DUSP5, involved in ERK1/2 pathway attenuation [54], and ARG2, whose over-expression has been associated with the thyroid cancer pathogenesis [55]. It would be also interesting to investigate whether the other members of the miR-199a-2/214 cluster (miR-199a-5p and miR-214), down-regulated by *RET/PTC1*, might cooperate in the regulation of the same networks.

A fraction of PTC, progressive and/or non-responding to radioiodine treatment, has a very poor prognosis, as there are no effective therapeutic options for them. MiRNA-driven therapy in cancer is still a challenging task for delivery, distribution and toxicity, but also because miRNA biology is still largely unknown [56]. Thus, a better understanding of both the signals that regulate miRNA expression and their functional impact in cancer cells is necessary to translate in a near future to clinics the use of miRNA as targets/tools.

In this line of research, our results unveiled miR-199a-3p as a novel player with tumor suppressor functions in the cancerogenesis of PTC. MiR-199a-3p, under-expressed in a consistent fraction of PTCs, may represent a potential tool to modulate macropinocytosis in thyroid tumor cells, hampering their survival.

## MATERIALS AND METHODS

### Cells cultures and pharmacological treatment

Primary thyrocyte cultures and thyrocytes expressing the *RET/PTC1* oncogene have been previously described [20]. T686 cells are a pool of clones derived from primary non-neoplastic human thyrocytes immortalized with SV40 large T antigen. T686 cells were selected in geneticin (G-418) (Invitrogen, Carlsbad, CA) supplemented Dulbecco's modified Eagle's medium (DMEM), Ham's F12 and MCDB (ratio 2:1:1; Invitrogen). The PTC-derived thyroid cell lines TPC1, NIM-1, BCPAP and K1 were provided by Dr. Santoro. The human cell lines A2780 (ovarian cancer) and HT-29 (colon cancer) were obtained from American Type Culture Collection (ATCC, Manassas, VA). Cells were cultured in DMEM (NIM-1, TPC1, and B-CPAP) or DMEM, Ham's F12, MCDB ratio 2:1:1 (K1) or RPMI-1640 (A2780) or McCoy's 5A (HT-29) (Invitrogen). All culture media were supplemented with 10% fetal bovine serum (FBS) (EuroClone) and cell were maintained at 37 °C and 5% CO2. Cell lines were authenticated by STR DNA Profiling Analysis using Stem Elite ID System Promega according to the manufacturer's instructions and ATCC guidelines.

RPI-1 synthesis and treatment of TPC1 cells have been described [21;30]. TPC1 cells were treated with RPI-1 (60 μM) for 24 hours in complete medium.

Vemurafenib (PLX4032) (Selleck Chemicals, Houston, TX) was used as a selective BRAFV600E inhibitor. NIM-1 cells were treated with vemurafenib (1 μM) for 20 hours in culture medium containing 2,5% FBS as described [31]. After treatment, RNA or total protein were obtained for TPC1 and NIM-1 cells.

Staurosporine (Sigma-Aldrich) was used for inducing cell death via intrinsic apoptotic pathways. K1 cells were treated with staurosporine (0,5 μM) for 24 hours. After treatment, total protein extraction and immunofluorescence analysis were performed.

Inhibitors were dissolved in DMSO used at final concentration of 0,1% (0,5% for RPI-1).

### Tissue specimens

Thyroid samples were collected at the Department of Pathology at Fondazione IRCCS Istituto Nazionale dei Tumori, Milano (INT). The PTCs (n=14) were classified according to WHO Classification [4] and the extension of disease according to pathological tumor-node-metastasis (pTNM) staging system [57]. The non-neoplastic thyroid tissues (n=7) were from patients with pathologies other than TC. Tumor sections were evaluated by a pathologist and representative tumor areas were selected.

Snap frozen tissue samples were analysed by qRT-PCR while FFPE (formalin fixed paraffin embedded) samples from the same patients were investigated by IHC analyses with the rabbit monoclonal antibody against MET (Ventana SP44 #790-4430) and using the BenchMark ULTRA ICH/ISH Staining Module, according to ULTRAView DAB detection kit and procedure.

For all samples informed consent, approved by the Independent Ethical Committee of the INT-MI, was obtained.

### RNA isolation

Total RNA with miRNA fraction was extracted with miRNeasy mini kit (Qiagen, Hilden, Germany); for gene expression analysis, RNA was further purified using the RNeasy MinElute Cleanup Kit (Qiagen).

### MiRNAs/ genes microarray profiling

Gene expression profiles for model-1 have been previously reported (ArrayExpress accession no. E-MEXP-429) [20] and now updated. MiRNA profiles for both models and gene profiles for model-2 have been obtained by using Illumina platforms and were deposited on NCBI's Gene Expression Omnibus (GEO) database, superSeries GSE49415. RNA samples were processed for microarray hybridization by the INT-MI Functional Genomics and core facility as previously described [58].

Further details about RNA samples processing, data analysis, miRNA targets prediction, integrated analysis and gene ontology are provided in Supplementary Methods.

### qRT-PCR

Expression levels of mature miRNAs were quantified by two-step quantitative real-time PCR.

Fifty nanograms of RNA were reverse transcribed with TaqManMicroRNA Reverse Transcription Kit (Applied Biosystems Inc., Foster City, CA). Quantification was performed with TaqMan miRNA assays (Applied Biosystems). U6-snRNA and let-7a were selected as endogenous controls for RNA input normalization based on their high expression and low variability among our samples. Gene expression levels were assessed as described [27], HPRT was used as housekeeping gene.

All qRT-PCR reactions were performed in triplicate on ABI PRISM 7900HT Real-Time PCR System. Data were analysed with the SDS (Sequence Detection System) 2.4 and the RQ Manager 1.2.1 software, using the 2^−ΔΔCt^ method with a relative quantification RQmin/RQmax confidence set at 95%. The error bars display the calculated maximum (RQmax) and minimum (RQmin) expression levels that represent SEM of the mean expression level (RQ value). The upper and lower limits define the region of expression within which the true expression level value is likely to occur.

### Cells transfections

Thyroid cell lines were transfected with the synthetic miRNA mimic miR-199a-3p, (PM11779 Applied Biosystems) commercialy designed to mimic only the endogenous mature miRNA of interest, at 100 nM by siIMPORTER Transfection Reagent (Millipore, Billerica, MA). FAM-labeled Pre-miR Negative Control#1 and Pre-miR miRNA Precursor-Negative control#1 (Applied Biosystems) were used to monitor transfection efficiency and as non-targeting negative control.

MET and mTOR silencing was obtained by transfecting 10nM of specific siRNAs by Lipofectamine RNAiMAX (Invitrogen, Carlsbad, CA) following the manufacturer's reverse transfection protocol. All siRNA oligonucleotides (sequences in Supplementary Fig.S3A) were kindly provided by Nerviano Medical Sciences S.r.l.”. Commercial NON-TARGET small interfering RNA was used as control (ThermoScientific, Dharmacon Inc. Chicago, IL).

### Western blot analysis and antibodies

Total protein cell extracts, SDS PAGE and Western blot analyses were performed as described [27]. The primary antibody RET (C-19, #sc-167), pRET (Y1062, #sc-20252-R), Raf-1 (E-10, #sc-7267) and Met (C-12, #sc-10) are from Santa Cruz Biotechnology (Santa Cruz, CA). RET common II has been previously described [59]. ERK1/2, #M5670), pERK1/2, #M8159), βtubulin (#T4026), actin (#A2066) and vinculin (#V9131) are from Sigma-Aldrich. MEK1/2 (L38C12, #4694), pMEK (Ser217/221,#9121), Akt (#9272), pAkt (Ser473, #9271), mTOR (7C10, #2983), cleaved PARP (Asp214, #9541), and cleaved caspase-3 (Asp175, 5A1E, #9664) are from Cell Signaling Technology (Beverly, MA). Relative protein levels were quantified by the Quantity One 4.6.6 software (Bio-Rad, Hercules, CA).

### Cell proliferation, migration and viability assays

Cell proliferation was assessed using AlamarBlue assay (Invitrogen) as described [60]. Migration assay was performed in 24-well Transwell chambers (Costar, Corning, Inc., Corning, NY) as previously reported [54].

Cell viability was assessed by NucleoCounter™ system based on nuclei staining with propidium iodide (PI) (ChemoMetec A/S, Denmark) as described [61]. At the indicated times, cells, including their culture medium, were harvested and the percentage of cell death was calculated according to the manufacturer's procedures.

### Immunofluorescence

K1 cells treated with staurosporine or tranfected with miR-199a-3p and the relative controls were harvested and deposited onto slides by centrifugation at 250rpm for 15min using a Shandon Cytospin 2 Centrifuge. Cytospin-prepared slides were processed for immunofluorescence as described [27]. Cleaved caspase-3 was detected by monoclonal antibody followed by Alexa Fluor® 546 Goat Anti-Rabbit IgG (Invitrogen/Molecular Probes®). Slides were then coverslipped with ProLong® Gold Antifade Reagent with DAPI (P36935 Molecular Probes®).

### Macropinocytosis analysis

K1 cells were seeded in 8-well chamber-slides. 24h after seeding cells were transfected as described above. 70h after transfection cells were incubated with 1mg/ml FITC-Dextran (D-1820 Invitrogen/Molecular Probes®) in Opti-MEM® medium (Gibco Life technologies) at 37°C 5% CO2 for 1h. Following labeling, cells were PBS washed, fixed with 4% paraformaldehyde in PBS for 15min and coverslipped as described above.

### Statistical analyses

Statistical analyses and graphs were generated using GraphPad Prism version 5.0. For TCGA analysis p-values were calculated by a binomial negative distribution test using DESeq2 package of Bioconductor [62]. Other comparisons between two groups were performed by two-tailed Student's t test as stated in the figure legends. Statistical analyses applied on microarray data were detailed in Supplementary Methods.

## SUPPLEMENTARY FIGURES TABLE AND REFERENCES






