# Supplementary material for: miR-199a-3p displays tumor suppressor functions in papillary thyroid carcinoma
Source: Oncotarget. 2014 Mar 16;5(9):2513–28. doi: 10.18632/oncotarget.1830 (PMC4058023; doi:10.18632/oncotarget.1830)
Supplement: Supplementary file 1 [file oncotarget-05-2513-s001.pdf]

A

	ID (previous ID)	miRBase ACCESSION	CHR: start-end [strand] Nucleotides	Sequence
PRECURSOR	hsa-mir-199a-1	MI0000242	chr19 : 10928102-10928172 [-]	GCCAACCCAGUGUUCAGACUACCUGUUCAGGAGGCUCUCAAUGUGUACAGUAGUCUGCACAUUGGUUAGGC
MATURE	hsa-mir-199a-5p (hsa-mir-199a)	MIMAT0000231	6→28	CCCAGUGUUCAGACUACCUGUUC
	hsa-mir-199a-3p (hsa-mir-199a*)	MIMAT0000232	47→68	ACAGUAGUCUGCACAUUGGUUA
PRECURSOR	hsa-mir-199a-2 #	MI0000281	chr1 : 172113675-172113784 [-]	AGGAAGCUUCUGGAGAUCCUGCUCCGUCGCCCCAGUGUUCAGACUACCUGUUCAGGACAAUGCCGUUGUACAGUAGUCUGCACAUUGGUUAGACUGGGCAAGGGAGAGCA
MATURE	hsa-mir-199a-5p (hsa-mir-199a)	MIMAT0000231	31→53	CCCAGUGUUCAGACUACCUGUUC
	hsa-mir-199a-3p (hsa-mir-199a*)	MIMAT0000232	70→91	ACAGUAGUCUGCACAUUGGUUA
PRECURSOR	hsa-mir-199b	MI0000282	chr9: 131007000 -131007109 [-]	CCAGAGGACACCUCCACUCCGUCUACCCAGUGUUUAGACUAUCUGUUCAGGACUCCCAAAUUGUACAGUAGUCUGCACAUUGGUUAGGCUGGGCUGGGUUAGACCCUCGG
MATURE	hsa-mir-199b-5p (hsa-mir-199b)	MIMAT0000263	26→48	CCCAGUGUUUAGACUAUCUGUUC
	hsa-mir-199b-3p	MIMAT0004563	65→86	ACAGUAGUCUGCACAUUGGUUA

hsa-mir-199a-2 clustered with hsa-mir-214 :

PRECURSOR	hsa-mir-214	MI0000290	chr1: 172107938-172108047 [-]
MATURE	hsa-miR-214-5p (hsa-miR-214*)	MIMAT0004564	30→51
	hsa-miR-214-3p (hsa-miR-214)	MIMAT0000271	71→92

B

```
                                            1
hsa-miR-199a*_miRBase-9.1                   UACAGUAGUC UGCACAUUGG UU-
hsa-miR-199a*:9.1_Illumina                  UACAGUAGUC UGCACAUUGG ---
hsa-miR-199a-3p_miRBase-12                  -ACAGUAGUC UGCACAUUGG UUA
hsa-miR-199b-3p_miRBase-12                  -ACAGUAGUC UGCACAUUGG UUA
hsa-miR-199a-3p,hsa-miR-199b-3p_Illumina"   -ACAGUAGUC UGCACAUUGG UUA
```

Supplementary Figure S1. (A) Gene family miR-199. **(B)** hsa-miR-199a/b-3p probes sequences of Illumina humanMI V2 platform and the corresponding sequences deposited in miRBase version 9.1 and 12. Sequence alignment was generated using SeaView software v 4.4 [Gouy M., Guindon S. & Gascuel O. (2010) SeaView version 4 : a multiplatform graphical user interface for sequence alignment and phylogenetic tree building. *Molecular Biology and Evolution* 27(2):221-224.]

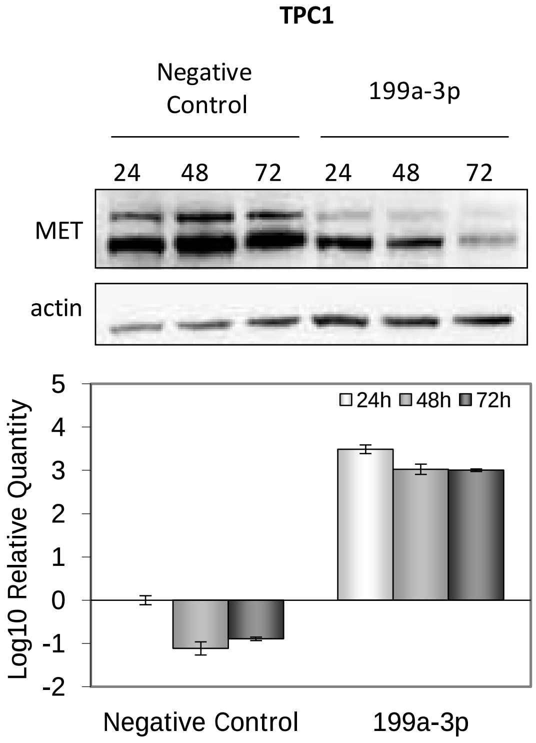

Supplementary Figure S2 TPC1 cell line transiently transfected with miR-199a-3p or Negative Control at the indicated time point. Total cell lysates were analysed by immunoblotting for MET expression using actin as loading control. Expression level of miR-199a-3p was measured by qRT-PCR, normalized to let-7a and expressed relative to negative control transfected cells at 24h. Results were presented as mean ± SEM from triplicate assays.

A

siRNA list

NTO ON TARGET plus <u>Non-Targeting Pool</u>
(Thermo Scientific) Catalog Item #D-001810-10

MET

13 5' GAA CAG AAU CAC UGA CAU A dTdT 3'
15 5' GUA AAU UGC GAU AAG GAA A dTdT 3'
17 5' CAA AGA AGG AAG UGU UUA A dTdT 3'
19 5' AGU GAG AGC ACG AUG AAU A dTdT 3'

mTOR

19 5' GGC CUA UGG UCG AGA UUU A dTdT 3'
21 5' GGU CUG AGU UUA AGG UCU A dTdT 3'
23 5' GCG CCA ACC UGG AUG ACU A dTdT 3'
25 5' GCU AUG UAG UAG AGC CCU A dTdT 3'

B

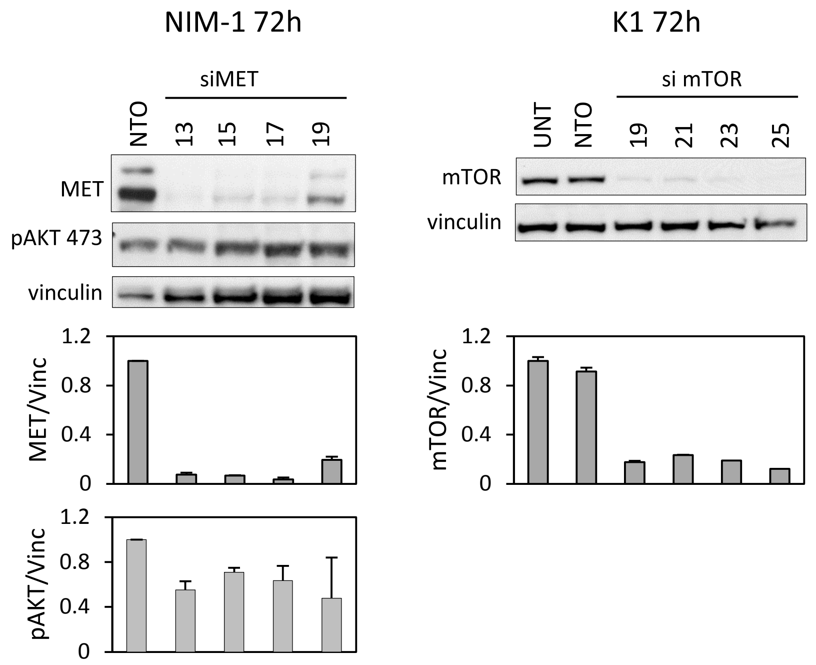

Supplementary Figure S3. (A) SiRNA sequences. **(B)** In set up experiments four different MET-specific and mTOR-specific siRNAs were assessed respectively in NIM-1 and K1 cells. Cells were left untransfected (UNT) or transfected with specific siRNAs (siMET or si mTOR) or Non-targeting control (NTO). 72h post-transfection total cell lysates were analysed by immunoblotting with the indicated antisera using vinculin as loading control. Protein levels were quantified by densitometric approach and represented relative to the value of Non-targeting control.

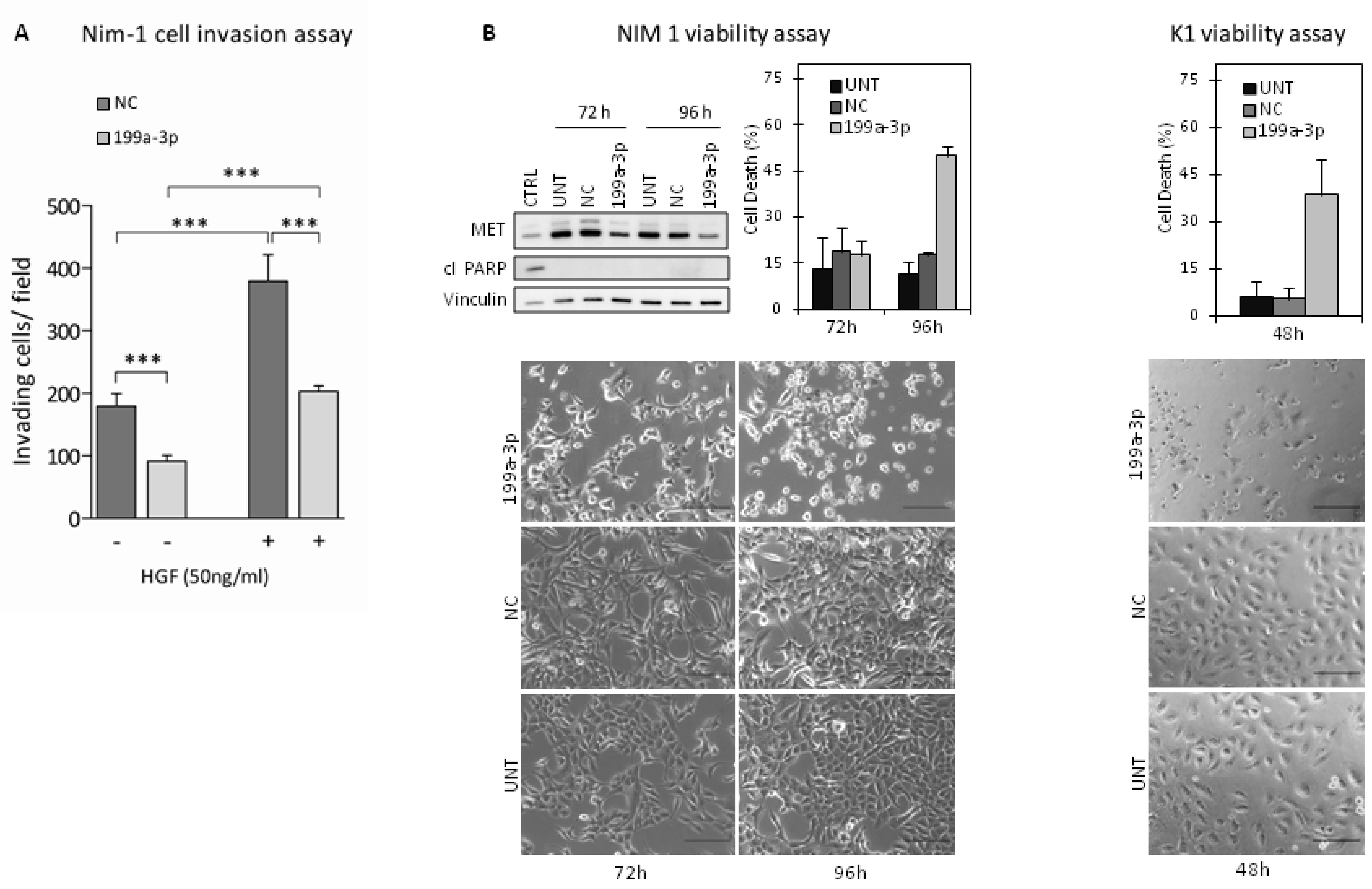

Supplementary Figure S4. (A) Invasion assay in NIM-1 cells transfected with miR-199a-3p (199a-3p) or Negative-Control (NC). Invasive capability was evaluated 48h post-transfection in Matrigel coated Transwell chambers in absence or presence of HGF. After 24 hours of incubation at 37°C, cells that invaded the Matrigel layer were fixed in 95% ethanol, stained with a solution of 0.4% SRB in 1% acetic acid, and counted under an inverted microscope. Data are reported as average cell number per field ± SD calculated as Migration assay Figure 4. ***P < 0.0001 statistical significance determined via Student's t-test. (B) NIM-1 and K1 cells were either left untransfected (UNT) or transfected with miR-199a-3p (199a-3p) or Negative-Control (NC) by Lipofectamine RNAiMAX (Invitrogen) transfection reagent at a final concentration of 100nM following the manufacturer's reverse transfection protocol. At the indicated times viability was assessed as described in Figure 5 and in Materials and Methods. Below representative images of NIM-1 and K1 cells at the indicated time points (LEICA inverted microscope, magnification 100x; Scale bar 200um)

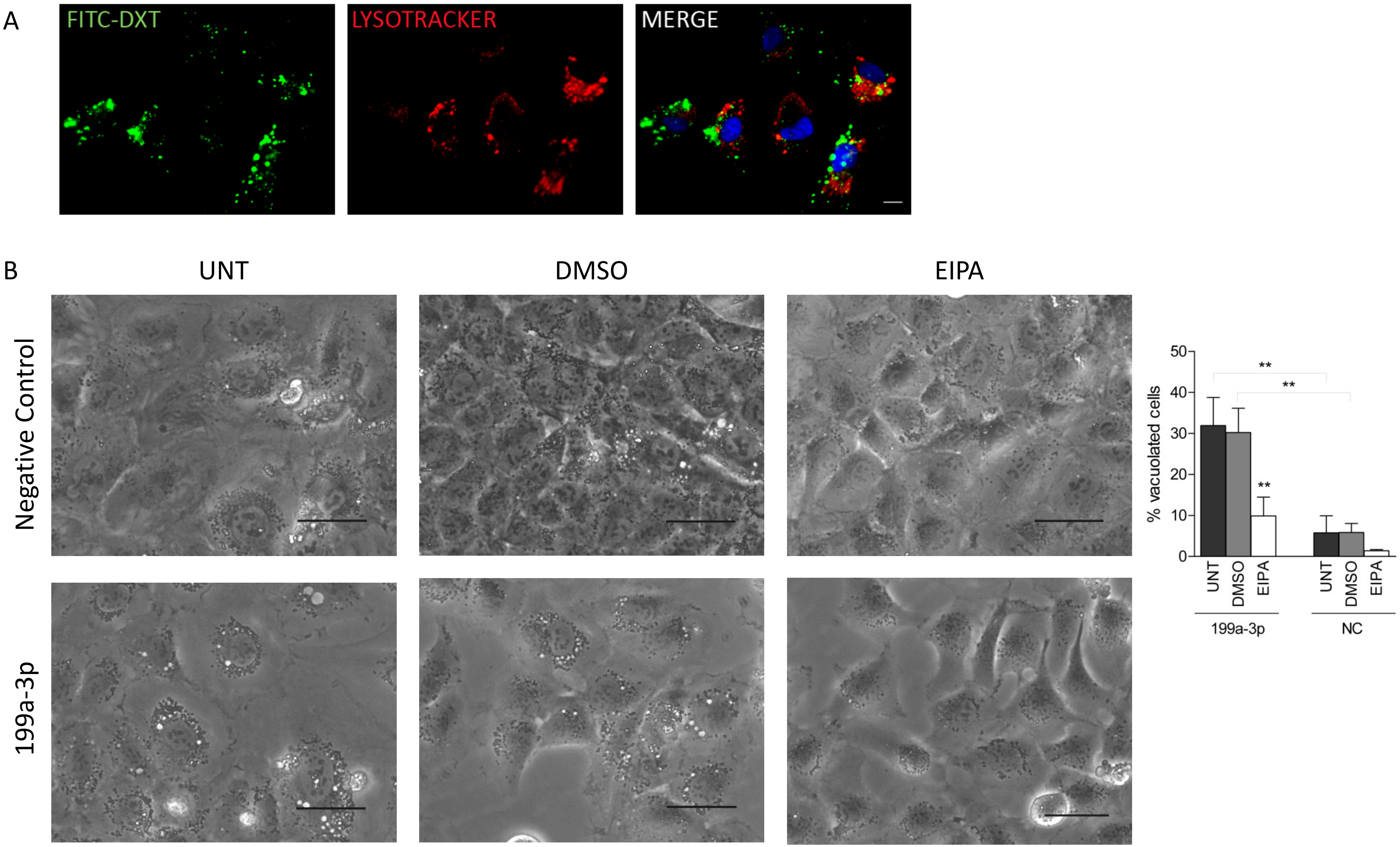

Supplementary Figure S5 **(A)** Representative images of K1 cells 72h post-transfection with miR-199a-3p. Cells were pre-incubated with 100nM Lysotracker Red DND-99 (L7528 Invitrogen/Molecular Probes®) in Opti-MEM® at 37°C. After 30min, 1mg/ml FITC-Dextran (Molecular Probes®) was added directly in the same medium and cells were colabeled at 37°C for additional 30min. Following staining cells were fixed and processed as described for macropinocytosis analysis in Material and Methods. Images were captured using a Nikon Eclipse Ti microscope and analyzed with Image-Pro Plus 7.0.1 software . Scale bar 20 µm.

(B)) Representative images of K1 cells 72h post-transfection with miR-199a-3p or Negative-Control, treated for 2 hours with either vehicle (DMSO) or 100µM EIPA or left untreated (UNT).
EIPA (5-(N-ethyl-n-isopropil)-amiloride from Sigma-Aldrich, St Louis, MO) was used as an inhibitor of macropinocytosis. Pictures were taken post treatment (LEICA inverted microscope, scale bar 50 µm). The percentage of the corresponding vacuolated cells were determined as described in Figure 7. **$P < 0.005$ statistical significance determined via Student's t-test.

Target	FC	DIANA	microRNA.org	mirDB	PITA	RNA22	TargetScan	Sum
C8orf4	31,2	X	X		X		X	4
AGPAT9	24,4	X	X				X	3
DUSP5	19,2	X			X	X		3
ITGA6	11,3	X	X		X			3
NTRK2	11,1				X		X	2
MAP3K5	7,6	X	X				X	3
ARG2	6,5	X	X				X	3
CDK17	6,4	X	X	X			X	4
PTPRU	6,3	X	X				X	3
TBX3	6,2	X	X				X	3
ITGA3	5,4	X	X		X	X	X	5
ARL4C	5,3	X			X			2
DCBLD2	5,3	X					X	2
NRIP1	5,1	X	X				X	3
NRP1	4,8				X	X		2
ABCA1	4,7	X			X			2
RIN2	4,4	X				X		2
CXADR	4,1			X			X	2
RBM47	4,0	X	X				X	3
PAQR3	3,9	X			X			2
C9orf72	3,8	X	X		X		X	4
MREG	3,7				X	X		2
TGIF1	3,7	X	X					2
GALNT7	3,6	X			X		X	3
NETO2	3,5	X			X			2
PAG1	3,5	X			X			2
ZNF217	3,4	X	X		X		X	4
ARHGEF3	3,3	X	X	X			X	4
OGFRL1	3,2	X	X		X		X	4
TRIM14	3,1	X				X		2
OAS3	3,1				X	X		2
GPR37	3,0	X	X				X	3
MAP3K4	3,0	X	X	X			X	4

Target	FC	DIANA	microRNA.org	mirDB	PITA	RNA22	TargetScan	Sum
ITGB8	3,0	X			X		X	3
PDE4B	2,9	X				X	X	3
NTNG1	2,9		X				X	2
MET	2,9				X	X		2
ERO1L	2,8	X	X				X	3
DSC2	2,7	X	X				X	3
CXCL12	2,7		X				X	2
LCOR	2,6	X					X	2
MAP4K4	2,5		X		X			2
SRPK1	2,5	X			X			2
JHDM1D	2,5	X				X		2
BCAR3	2,4	X	X	X			X	4
RTKN	2,4		X			X		2
TMEM107	2,4	X	X		X		X	4
SEMA3A	2,3	X	X				X	3
TAGLN3	2,3				X	X		2
PTPRE	2,3		X		X			2
TMEM117	2,3	X			X			2
MFHAS1	2,3		X			X		2
DPP4	2,2				X	X		2
ELMOD1	2,2	X			X			2
NOVA1	2,2	X	X	X			X	4
DKK3	2,2	X	X					2
PTPRZ1	2,2	X	X		X		X	4
TANC2	2,2				X	X		2
KIAA0319L	2,2	X	X				X	3
APLP2	2,1	X	X				X	3
CADPS	2,1		X		X		X	3
BAZ2B	2,1	X			X	X		3
SLC39A10	2,1	X	X		X	X	X	5
CXCL11	2,1	X	X		X		X	4
FAM84B	2,1	X				X		2
ATP2B1	2,0	X	X		X			3

Target	FC	DIANA	microRNA.org	mirDB	PITA	RNA22	TargetScan	Sum
SSFA2	2,0	X			X	X		3
FAM60A	2,0	X	X				X	3
SORBS2	2,0		X		X	X		3
BTN2A2	2,0	X			X			2
KIF2A	2,0	X			X		X	3
PLAG1	2,0	X			X		X	3
ZCCHC2	2,0	X			X	X		3
LPAR5	2,0	X	X				X	3
PLEKHH1	1,9	X				X	X	3
MFSD6	1,9	X	X					2
FBN2	1,9	X				X		2
GPR160	1,9	X	X	X			X	4
WSB1	1,9	X	X					2
GLI3	1,9	X			X			2
PRKX	1,9	X			X			2
ANKRD57	1,9		X				X	2
BCAP29	1,9	X			X			2
OSBPL3	1,9	X			X			2
UXS1	1,9	X	X					2
TMED5	1,9	X	X	X	X		X	5
YES1	1,9		X				X	2
CHD2	1,9	X	X		X	X		4
NLK	1,9	X	X				X	3
RPAP3	1,8	X					X	2
PPM1H	1,8	X			X			2
TRIM36	1,8	X	X				X	3
MCC	1,8	X			X			2
ABI1	1,8	X				X		2
FGD6	1,8	X			X			2
PDP1	1,8	X	X					2
CD2AP	1,8		X	X			X	3
RAB3D	1,8					X	X	2
CHMP5	1,8	X	X	X			X	4

Target	FC	DIANA	microRNA.org	mirDB	PITA	RNA22	TargetScan	Sum
HSDL1	1,8	X	X		X		X	4
FAM96A	1,7	X			X			2
NRK	1,7	X	X		X			3
TXLNG	1,7	X	X				X	3
PON2	1,7	X	X	X	X		X	5
STAG2	1,7	X				X		2
EYA4	1,7	X			X			2
PTPRJ	1,7		X		X	X		3
LYPLA1	1,7	X	X		X			3
DTNA	1,7		X		X			2
SCARB2	1,7	X			X			2
SNN	1,7	X			X		X	3
KIAA0907	1,7	X	X				X	3
NANOS1	1,6				X		X	2
TMEM62	1,6	X	X				X	3
C4orf32	1,6	X					X	2
USP3	1,6		X		X			2
TNKS	1,6	X	X		X			3
RAP2B	1,6	X			X			2
NEDD4	1,6		X		X		X	3
TAB3	1,6	X			X			2
TANC1	1,6	X	X				X	3
AVPI1	1,5	X	X				X	3
FXR1	1,5	X	X	X			X	4
RB1	1,5	X	X				X	3
PEG10	1,5				X	X		2
AGPAT4	1,5				X	X		2
COL4A5	1,5	X	X		X		X	4
ZBTB33	1,5		X		X			2
NINL	1,5	X	X				X	3
UBXN2B	1,5	X					X	2
AUTS2	1,5				X		X	2
RRM2B	1,5	X	X				X	3
MGEA5	1,5	X	X				X	3
GPRASP1	1,5	X			X			2

Supplementary Figure S6. Inversely correlated targets of miR-199a-3p . The list was generated by integrating miR-199a-3p targets genes predicted by at least 2 algorithms with differentially expressed genes of model 1 (see Supplementary Materials and Methods). Target genes are reported following decreasing Fold Change (FC) order.
