# Supplementary material for: miR-199a-3p displays tumor suppressor functions in papillary thyroid carcinoma
Source: Oncotarget. 2014 Mar 16;5(9):2513–28. doi: 10.18632/oncotarget.1830 (PMC4058023; doi:10.18632/oncotarget.1830)
Supplement: Supplementary file 2 [file oncotarget-05-2513-s002.docx]

TABLE 1 Clinicopathologic characteristics of PTC/PDTC tumors samples analysed for miR-199a -3p qRT-PCR and for MET IHC

| **PATIENT ID** | **gender** | **age** | **diagnosis** | **histologic subtype** | **T** | **N** | **M** | **stage** | **thyroiditis** |
| --- | --- | --- | --- | --- | --- | --- | --- | --- | --- |
|  |  |  |  |  |  |  |  |  |  |
| PTC 1 | M | 46 | PTC | conventional | 3 | 1A | 0 | 3 | no |
| PTC 2 | F | 33 | PTC | conventional | 3 | 1B | 0 | 1 | yes |
| PTC 3 | F | 38 | PTC | conventional | 3m | 1B | 0 | 1 | yes |
| PTC 4 | F | 48 | PDTC | tall cell | 3 | 1A | 0 | 3 | yes |
| **PTC 5** | F | 52 | PTC | follicular | 3 | X | 0 | 3 | yes |
| PTC 6 | M | 35 | PTC | conventional | 3m | 1A | 0 | 1 | yes |
| PTC 7 | F | 38 | PTC | conventional | 3 | X | 0 | 1 | yes |
| PTC 8 | F | 39 | PTC | follicular | 3 | X | 0 | 1 | no |
| PTC 9 | M | 52 | PDTC | other | 3m | X | 0 | 3 | no |
| **PTC 10** | F | 61 | PDTC/PTC | tall cell | 2m | 1A | 0 | 3 | yes |
| **PTC 11** | F | 51 | PTC | follicular | 3 | 0 | 0 | 3 | yes |
| PTC 12 | F | 75 | PTC | tall cell | 3m | 1A | 1 (L, B, S) | 4C | yes |
| **PTC 13** | F | 51 | PDTC | tall cell | 3 | 0 | 0 | 2 | no |
| PTC 14 | M | 36 | PTC | follicular | 3 | X | 0 | 1 | yes |

m, multifocal; L, lung, B brain, S skin metastasis; pTNM, pathological tumor-node-metastasis staging; Nx, regional lymph nodes cannot be assessed
