# Supplementary material for: miR-199a-3p displays tumor suppressor functions in papillary thyroid carcinoma
Source: Oncotarget. 2014 Mar 16;5(9):2513–28. doi: 10.18632/oncotarget.1830 (PMC4058023; doi:10.18632/oncotarget.1830)
Supplement: Supplementary file 3 [file oncotarget-05-2513-s003.pdf]

Gene expression and miRNAs profiling

RNA samples were processed for mRNA microarray hybridization by the INT-MI Functional Genomics and core facility, as previously described [1]. Briefly, 800 ng of total RNA was reverse transcribed, labeled with biotin and amplified overnight (14 hours) using the Illumina RNA TotalPrep Amplification kit (Ambion, Austin, Texas, USA) according to the manufacturer's protocol. One µg of the biotinylated cRNA sample was mixed with the Hyb E1 hybridization buffer containing 37.5% (w/w) formamide and then hybridized to HumanHT-12 v3 Expression BeadChip (Illumina, Inc., San Diego, CA) at 58° C overnight (18 hours). The array represents over 48800 bead types, each with a unique sequence derived from human genes in the National Centre for Biotechnology Information Reference Sequence or UniGene database. Array chips were washed with the manufacturer's E1BC solution, stained with 1 µg/ml Cy3-streptavidine (Amersham Biosciences; GE Healthcare, Piscataway, NJ, USA) and eventually scanned with Illumina BeadArray Reader V3.1.3.0.

Mature miRNAs were amplified with the Human v2 MicroRNA Expression Profiling Kit based on the DASL (cDNA-mediated Annealing, Selection, Extension, and Ligation) assay, according to the manufacturer's instructions. Briefly, 600ng total RNA was converted to cDNA and annealed to a miRNA-specific oligonucleotide pool consisting of three parts: a universal PCR priming site at the 5' end, an address sequence complementary to a capture sequence on the BeadArray, and a miRNA-specific sequence at the 3' end. After PCR amplification and fluorescent labeling, the probes were hybridized on Illumina humanMI V2 arrays. After hybridization and washing, fluorescent signals were detected by the Illumina BeadArray Reader V3.1.3.0.

Microarray data analysis

Affymetrix gene expression data of model-1 were pre-processed using *rma* [2] method implemented in the *affy* [3] package of Bioconductor [4]. After normalization, probesets called "absent" in at least two samples were filtered out from subsequent analysis.

All data from Illumina gene expression and miRNA platforms were analyzed using the *lumi* package [5]. Data were log2 transformed and normalized using robust spline normalization.

Boxplot, MA-plot and between-sample correlation were used to evaluate the quality of array profiles. Two samples (one treated and one control) from the miRNA dataset of model-2 were excluded due to low quality profiles.

In each experiment, probes with a detection p-value > 0.01 in all samples were filtered out.

Finally, for genes represented by multiple probes, the probe detected in the largest number of samples (according to present/absent call or detection p-value) was chosen as the representative one.

In order to identify differentially expressed mRNAs and miRNAs, we performed a class comparison analysis, as implemented in the *limma* package [6], except for miRNA profiles of model-1 as no biological replicates were available. In this case, we performed only a fold-change analysis, aware of the higher risk of false positive hits. In order to reduce this risk, we filtered out miRNAs with an expression value < 8 in both samples.

All p-values were adjusted for multiple testing using Benjamini Hochberg false discovery rate (FDR) [7].

Identification of miRNA targets and integration with expression data

In silico prediction of miRNA targets was performed with 6 algorithms simultaneously (DIANA MicroT-CDS [8], microRNA.org [9] database based on miRanda algorithm [10], mirDB [11;12], PITA [13], RNA22 [14], TargetScan v6.2 [15]) using the HUGO gene symbol as common identifier. Conversion of ENSEMBL Transcript IDs to gene symbols was performed with *biomaRt* package version 2.14.0 [16]. We filtered the output of each algorithm, discarding those targets with a prediction score < 75th percentile of its score distribution and, finally, a gene was selected as putative target if it was predicted by at least two algorithms.

To support computational target prediction with experimental data, we integrated target prediction output with gene expression data, selecting those targets with a statistically significant modulation in the opposite direction of the particular miRNA under investigation.

Gene Ontology and pathway analysis

Gene Ontology [17] over-representation analysis was performed separately on the lists of commonly up- and down- regulated genes between the two models, using the *topGO* package [18]. Only terms associated to the biological process domain with a number of genes between 15 and 300 were tested. Resulting p-values from classical Fisher's exact test were corrected with Benjamini Hochberg false discovery rate and terms with an FDR < 0.01 were selected. Deregulated targets of hsa-miR-199a-3p were also investigated using IPA (Ingenuity® Systems, www.ingenuity.com).

Reference List

1. Callari M, Dugo M, Musella V, Marchesi E, Chiorino G, Grand MM, Pierotti MA, Daidone MG, Canevari S, De CL. Comparison of microarray platforms for measuring differential microRNA expression in paired normal/cancer colon tissues. PLoS One 2012;7:e45105.

2. Irizarry RA, Bolstad BM, Collin F, Cope LM, Hobbs B, Speed TP. Summaries of Affymetrix GeneChip probe level data. Nucleic Acids Res 2003;31:e15.

3. Gautier L, Cope L, Bolstad BM, Irizarry RA. affy--analysis of Affymetrix GeneChip data at the probe level. Bioinformatics 2004;20:307-315.

4. Gentleman RC, Carey VJ, Bates DM, Bolstad B, Dettling M, Dudoit S, Ellis B, Gautier L, Ge Y, Gentry J, Hornik K, Hothorn T, Huber W, Iacus S, Irizarry R, Leisch F et al. Bioconductor: open software development for computational biology and bioinformatics. Genome Biol 2004;5:R80.

5. Du P, Kibbe WA, Lin SM. lumi: a pipeline for processing Illumina microarray. Bioinformatics 2008;24:1547-1548.

6. Smyth GK. Linear models and empirical bayes methods for assessing differential expression in microarray experiments. Stat Appl Genet Mol Biol 2004; 3:Article3.

7. Benjamini Y and Hochberg Y. Controlling the false discovery rate: a practical and powerful approach to multiple testing Journal of the Royal Statistical Society, Series *B* 1995; 57 (1): 289–300

8. Reczko M, Maragkakis M, Alexiou P, Grosse I, Hatzigeorgiou AG. Functional microRNA targets in protein coding sequences. Bioinformatics 2012;28:771-776.

9. Betel D, Wilson M, Gabow A, Marks DS, Sander C. The microRNA.org resource: targets and expression. Nucleic Acids Res 2008;36:D149-D153.

10. Enright AJ, John B, Gaul U, Tuschl T, Sander C, Marks DS. MicroRNA targets in Drosophila. Genome Biol 2003;5:R1.

11. Wang X, El N, I. Prediction of both conserved and nonconserved microRNA targets in animals. Bioinformatics 2008;24:325-332.

12. Wang X. miRDB: a microRNA target prediction and functional annotation database with a wiki interface. RNA 2008;14:1012-1017.

13. Kertesz M, Iovino N, Unnerstall U, Gaul U, Segal E. The role of site accessibility in microRNA target recognition. Nat Genet 2007;39:1278-1284.

14. Miranda KC, Huynh T, Tay Y, Ang YS, Tam WL, Thomson AM, Lim B, Rigoutsos I. A pattern-based method for the identification of MicroRNA binding sites and their corresponding heteroduplexes. Cell 2006;126:1203-1217.

15. Lewis BP, Burge CB, Bartel DP. Conserved seed pairing, often flanked by adenosines, indicates that thousands of human genes are microRNA targets. Cell 2005;120:15-20.

16. Durinck S, Spellman PT, Birney E, Huber W. Mapping identifiers for the integration of genomic datasets with the R/Bioconductor package biomaRt. Nat Protoc 2009;4:1184-1191.

17. Ashburner M, Ball CA, Blake JA, Botstein D, Butler H, Cherry JM, Davis AP, Dolinski K, Dwight SS, Eppig JT, Harris MA, Hill DP, Issel-Tarver L, Kasarskis A, Lewis S, Matese JC et al. Gene ontology: tool for the unification of biology. The Gene Ontology Consortium. Nat Genet 2000;25:25-29.

18. Alexa A, Rahnenfuhrer J, Lengauer T. Improved scoring of functional groups from gene expression data by decorrelating GO graph structure. Bioinformatics 2006;22:1600-1607.
